# Antimicrobial Bilayer Nanocomposites Based on the Incorporation of As-Synthetized Hollow Zinc Oxide Nanotubes

**DOI:** 10.3390/nano10030503

**Published:** 2020-03-11

**Authors:** Carol López de Dicastillo, Cristian Patiño Vidal, Irene Falcó, Gloria Sánchez, Paulina Márquez, Juan Escrig

**Affiliations:** 1 Center of Innovation in Packaging (LABEN), Department of Food Science and Technology, Technology Faculty, University of Santiago de Chile (USACH), Santiago 9170201, Chile; cristian.patino@usach.cl; 2 CEDENNA (Center for the Development of Nanoscience and Nanotechnology), Santiago 9170124, Chile; paulina.marquez.m@usach.cl (P.M.); juan.escrig@usach.cl (J.E.); 3 Department of Preservation and Food Safety Technologies, Institute of Agrochemistry and Food technology (IATA-CSIC), 46980 Paterna, Valencia, Spain; iff26388@hotmail.es (I.F.); gloriasanchez@iata.csic.es (G.S.); 4 Department of Physics, University of Santiago de Chile (USACH), Santiago 9170124, Chile

**Keywords:** atomic layer deposition, electrospinning, nanotube, zinc oxide

## Abstract

An antimicrobial polymeric bilayer structure based on the application of an acrylic coating containing hollow zinc oxide nanotubes over a polymeric substrate was developed in this work. Firstly, zinc oxide nanotubes (ZnO_NT_) were obtained by an atomic layer deposition (ALD) process over electrospun polyvinyl alcohol nanofibers followed by polymer removal through calcination with the purpose of obtaining antimicrobial nanostructures with a high specific area. Parameters of electrospinning, ALD, and calcination processes were set in order to obtain successfully hollow zinc oxide nanotubes. Morphological studies through scanning electron microscopy (SEM) and transmission electron microscopy (TEM) microscopies confirmed the morphological structure of ZnO_NT_ with an average diameter of 180 nm and thickness of approximately 60 nm. Thermal and X-ray diffraction (XRD) analyses provided evidence that calcination completely removed the polymer, resulting in a crystalline hexagonal wurtzite structure. Subsequently, ZnO_NT_ were incorporated into a polymeric coating over a polyethylene extruded film at two concentrations: 0.5 and 1 wt. % with respect to the polymer weight. An antimicrobial analysis of developed antimicrobial materials was performed following JIS Z2801 against *Staphylococcus aureus* and *Escherichia coli.* When compared to active materials containing commercial ZnO nanoparticles, materials containing ZnO_NT_ presented higher microbial inhibition principally against Gram-negative bacteria, whose reduction was total for films containing 1 wt. % ZnO_NT_. Antiviral studies were also performed, but these materials did not present significant viral reduction.

## 1. Introduction

Nowadays, the interest toward antimicrobial materials, such as antimicrobial packaging, surfaces, and paintings, has increased due to the high incidence of nosocomial diseases, hospital infections, and the increase of microbial resistance. Several developments based on the incorporation of antimicrobial substances into polymeric matrixes have been carried out in order to reduce and inhibit microbial presence [1,2]. Specifically, the research focused on novel antimicrobial nanocomposites whose activities are related to antimicrobial nanoparticles (NPs) has increased over last years since antimicrobial nanostructures are one of the most promising alternatives in the search for new antimicrobial substances [3,4,5]. The attention has been principally centered on metallic/metal oxide nanoparticles due to their broad antibacterial and antifungal activities at low concentrations thanks to their high specific area [6,7,8,9].

Zinc oxide (ZnO) nanoparticles are an inorganic material with optical, chemical sensing, electric conductivity, catalytic, photochemical, and antimicrobial properties. This metal oxide presents three crystal structures: wurtzite, zinc blend, and rocksalt, with a direct wide band gap of 3.3 Ev [10]. Some works have evidenced ZnO nanoparticles with dimensions smaller than 100 nm have shown increasing antimicrobial properties against Gram-negative and Gram-positive bacteria due to a higher cellular internalization. Specifically, Verma et al. (2018) studies have indicated that the antibacterial activity of ZnO nanoparticles against *Staphylococcus aureus* and *Escherichia coli* increased with the decrease in their size from 250 to 80, 40, and 20 nm due to an increased reactive oxygen species generation and membrane damage in bacteria [11,12]. Different synthesis methods as sol–gel, hydrothermal, simple thermal sublimation, vapor–liquid–solid, double-jet precipitation, self-combustion, and “green synthesis” have been used to obtain ZnO nanorods, nanospheres, nanotubes, nanowires, and nanoneedles [13,14,15]. The control of synthesis conditions and parameters is very important in order to obtain homogeneous NPs, since antimicrobial activity is highly influenced by NPs’ morphology, structure, and shape. Thus, in this work, a new controlled methodology to synthesize homogenous antimicrobial hollow ZnO nanotubes was developed based on the combination of electrospinning and atomic layer deposition technologies. Electrospinning (EP) is an efficient, simple, cost-effective, and scalable technique to generate polymeric fibers. The obtaining of the fibers occurs when a voltage is applied to the polymer solution, obtaining a flow with a conical structure known as the “Taylor Cone”, where the solvent evaporates during the process to finally obtain thin structures with a high specific area [16,17]. On the other hand, the atomic layer deposition (ALD) is a technique that allows the fabrication of nanometric layers of a great variety of materials in different substrates [18]. This technique consists in the generation of thin films by introducing sequentially and cyclically two or more precursors in gas phase, under conditions of controlled temperature and pressure, where the precursors react chemically with the surface of a substrate, allowing the formation of atomic-scale monolayers [18,19]. Generally, a metal–organic precursor and a co-reactant as an oxygen source or as reducing agent are used [20]. One of the advantages that most attracts the attention of this technique is the ability to generate a unique uniform deposition, which allows the thickness of the film to be controlled by the amount of ALD cycles and, in addition, complex three-dimensional surfaces can be uniformly coated. These characteristics give it a great versatility that have allowed its application in a wide variety of areas, such as microelectronics, energy storage systems, water treatment, catalysis, and medicine and biology [21,22,23,24,25,26,27,28,29]. Nevertheless, the combination of EP and ALD has been scarcely used to synthesize nanoparticles with antimicrobial purposes. Furthermore, an antimicrobial bilayer structure was developed by applying a polymeric coating containing synthesized ZnO nanotubes over a polymeric substrate.

## 2. Materials and Methods

### 2.1. Polymers, Chemicals, and Microorganisms

Diethylzinc (Zn(C_2_H_5_)_2_, DEZn, min. 95%), in a 50 mL Swagelok^®^ cylinder was purchased from Strem Chemicals Inc. (Newburyport, MA, USA). Gohsenol AH-17 polyvinyl alcohol (PV) (saponification degree 97–98.5% and viscosity 25–30 mPa.s) was obtained from The Nippon Synthetic Chemical Co. (Osaka, Japan). Zinc oxide nanoparticles (ZnONP) were purchased from Sigma-Aldrich. Commercial acryl-based coating Elvacite 2010 was purchased from Lucite International (Chile).

Gram-negative *Escherichia coli* ATCC 25,922 and Gram-positive and *Staphylococcus aureus* ATCC 25,923 bacteria were chosen as bacteria strain models. Bacterial strains were obtained from Biotechnology and Applied Microbiology Laboratory (LAMAP) (Santiago, Chile) and stored in glycerol 30% at −80 °C until needed. For experimental use, the stock cultures were maintained in tryptone soy agar slants at 4 °C and transferred monthly. Prior to each experiment, a loopful of each strain was transferred to 5 mL of tryptone soy agar and incubated at 37 °C for 16 h to obtain fresh early-stationary phase cells.

The cytopathogenic F9 strain of Feline calicivirus (FCV, ATCC VR-782) was propagated and assayed on CRFK (Crandell Reese Feline Kidney) cells from ATCC (CCL-94). Semi-purified stocks were subsequently produced on CRFK cells by the centrifugation of infected cell lysates at 660× g for 30 min after 2–3 days post-infection. Infectious viruses were calculated by determining the 50% tissue culture infectious dose (TCID50) using the Spearmen–Karber method [30].

### 2.2. Development of ZnO Hollow Nanotubes (ZnO_NT_)

Electrospun poly(vinyl alcohol) nanofibers (PV_f_) were developed by using an electrospinning system (Spraybase^®^power Supply Unit, Maynooth, Ireland), according to our previous work [31]. Poly(vinyl alcohol) solution at 8% (*w/v*) was prepared in distilled water with stirring at 90 °C. The polymeric solution was transferred in a 5 mL plastic syringe and connected through a polytetrafluoroethylene tube to a stainless steel needle of 1.6 mm diameter charged by a high-voltage power supply with a range of 0–20 kV. Finally, PV_f_ nanofibers were obtained using 1.5 mL/h as the flow rate and 10 cm as the distance between the tip needle and the collector.

Subsequently, PV_f_ nanofibers behaved as a template for the preparation of hollow ZnO_NT_. Zinc oxide was deposited over PV_f_ fibers using a Savannah S100 ALD reactor (Ultratech, San Jose, CA, USA) maintained at 150 °C. Water (H_2_O) and diethylzinc (Zn(C_2_H_5_)_2_, DEZn) were used as precursors. The atomic layer deposition (ALD) pulse lengths were set at 0.12 s for DEZn dosing, 3 s for N_2_ purging, 0.15 s for H_2_O dosing, and 3 s for N_2_ purging [32]. Deposition based on 300 and 500 cycles was performed in order to investigate the effect of the number of ALD cycles on the thickness and quality of the coating.

Lastly, two methodologies were tested to remove the polymer from the inner zone of the metal oxide-deposited nanofibers. (i) Process A: ZnO-PV_f_ suffered a thermal treatment process in order to remove PV polymer from the inner side. Deposited nanofibers were calcinated at 450 °C and 600 °C during 1 h at air atmosphere. (ii) Process B: Thanks to the hydrosoluble nature of PV polymer, an alternative polymeric removal process was attempted by washing ZnO-deposited nanostructures with hot distilled water. Samples were sonicated into distilled water at 90 °C for 1 h. The sample that resulted from the washing process was named (ZnO-PV_f_)-B.

### 2.3. Characterization of ZnO Hollow Nanotubes

During the three stages for obtaining hollow ZnO_NT_, each structure was observed using scanning electron microscopy, SEM (Zeiss EVO MA10 SEM, Oberkochen, Germany), at 20 kV. Developed ZnO_NT_ were also analyzed through transmission electron microscopy, TEM (Hitachi HT7700 high resolution TEM, Chiyoda, Tokyo, Japan), at 100 kV.

X-ray Diffraction (XRD) patterns were measured using a Siemens diffractometer D5000 (Siemens AG, Erlangen, Germany) (30 mA and 40 kV) using Cu Ka (λ = 1.54 Å) radiation at room temperature. All scans were performed in a 2θ range of 2–80° at 0.02° s^−1^.

The small-angle X-ray scattering (SAXS) measurement was performed using a Bruker N8 Horizon model equipped with a Cu source (radiation Kα_1_, λ = 1.54060 Å, radiation Kα_2_, λ = 1.54439 Å) and 2D VÅNTEC-500 detector and MONTEL optics (Bruker AXS GmbH, Karlsruhe, Germany). The data were acquired in the q-range from 0.012 to 0.37 Å^−1^ with a measurement time of 7200 s in vacuum (2 mbar) and at room temperature. The generator was operated at 1 kW (40 kV and 25 mA). The data processing and analysis were performed using a Bruker DIFFRAC.SAXS program (Bruker AXS GmbH, Karlsruhe, Germany) that can fit and evaluate the size of the structures assuming different geometries.

Thermogravimetric analyses (TGA) were carried out through a Mettler Toledo Gas Controller GC20 Stare System TGA/DCS (Schwerzenbach, Switzerland). First, 5–6 mg of each sample was heated from 30 to 600 °C at 10 °C/min under nitrogen atmosphere with a flow rate of 50 mL min^−1^.

### 2.4. Development of Antimicrobial Bilayer Systems Containing ZnO Nanoparticles

Two antimicrobial bilayer structures were obtained by coating acrylic solutions containing zinc oxide nanotubes (ZnO_NT_) at 0.5 and 1 wt % with respect to acrylic polymer over an extruded 32 µm-polyethylene (PE) substrate film. According to the ZnO_NT_ concentrations, the bilayer structures were named 0.5ZnO_NT_-Acry/PE and 1ZnO_NT_-Acry/PE. ZnO_NT_ were dispersed in the 20 wt % acrylic solution under ethyl acetate. Before the coating process, a corona treatment on PE films was carried out by using a FT-350 mm Model equipment (HT, Taiwan) to improve coating adherence. Coatings were performed with a RK-Print multicoater equipment model K303 (RK Print Coat Instruments Ltd., Litlington, UK) by applying 6 mL of each solution onto PE films using a stainless steel rod No. 4 at 5 m min^−1^. Finally, coatings were dried during one minute at room temperature, and their thickness values were approximately 3–4 μm. In order to compare the antimicrobial effectiveness of ZnO_NT_-containing films, bilayer structures with commercial zinc oxide nanoparticles (ZnONP) were also analyzed (the TEM micrograph of ZnONP can be found in Figure S3 in Supplementary Material). These bilayer structures were obtained following the same method described above, and films were named 0.5ZnONP-Acry/PE and 1ZnONP-Acry/PE, when containing 0.5 and 1 wt % of ZnONPs, respectively.

### 2.5. Scanning Electronic Microscopy (SEM) Characterization of Bilayer Structure

SEM micrographs of the bilayer structure containing the highest concentration of ZnO_NT_ were performed through a SEM (Zeiss EVO MA10 SEM) scanning microscope with an accelerating voltage at 10 kV. Bilayer films were firstly cryofractured by previous immersion into liquid nitrogen. Subsequently, films were coated with gold palladium with a Anatech Hummer 6.2 sputtering system (Anatech USA, Sparks, USA). SEM micrographs of the cross-sections of the films were taken.

### 2.6. Antibacterial Activity of Bilayer Nanocomposites

The antibacterial activities of ZnO_NT_-Acry/PE and ZnONP-Acry/PE bilayer films were tested against Gram-negative bacteria *Escherichia coli* and Gram-positive bacteria *Staphylococcus aureus* according the Japanese Industrial Standard JIS Z 2801:2000 [33]. Test inoculums of each microorganism with a final concentration of 10^5^ CFU mL^−1^ were prepared. Film samples were prepared cutting coated and uncoated PE films in squares of 5 cm × 5 cm, while cover films were prepared cutting uncoated PE films in squares of 4 cm × 4 cm. Three specimens of each test sample and cover films were prepared and sterilized with 70% ethanolic solution. Test samples were collocated in a sterile Petri dish maintaining the coated surface exposed outside, and 400 µL of test inoculums were pipetted over them. Cover films were collocated upon test samples, avoiding the leak of test inoculums by edges, and the Petri dishes covered with the lid were incubated at 35 °C and of 93.2% relative humidity (RH) for 24 h. The test inoculum of test samples was recovered by manually stirring 10 mL of sterile phosphate buffer added in each Petri dish, and 10-fold dilutions with PBS were done. Then, 1 mL of each dilutions was placed in sterile Petri dishes and 20 mL of tryptic soy agar was poured in order to count the viable cells after incubating the Petri dishes at 35 °C for 48 h.

### 2.7. Determination of Virucidal Activity

Determination of the virucidal activities of films containing 0.5 and 1% wt. % of ZnO nanostructures were performed by adapting the standard. Briefly, 50 µL of FCV suspension diluted in PBS (phosphate buffer solution) at 3 and 6 log TCID_50_/mL was placed onto the test films (3 cm × 3 cm) and covered by a piece of low-density polyethylene (LDPE) (2.5 cm × 2.5 cm). Samples were stored overnight at 25 °C at 100% RH. Thereafter, the top film was lifted, and the virus droplet-exposed sides were recovered and 10-fold diluted with PBS. Then, infectious viruses were enumerated by cell culture assay as described above. Each treatment was done in triplicate. The virus decay titer was calculated as log_10_ (N_x_/N_0_), where N_0_ is the infectious virus titer for untreated films and N_x_ is the infectious virus titer for treated films [30].

## 3. Results and Discussion

### 3.1. Morphological Characterization of ZnO_NT_ and Nanocomposites Containing ZnO_NT_

**Figure 1:**
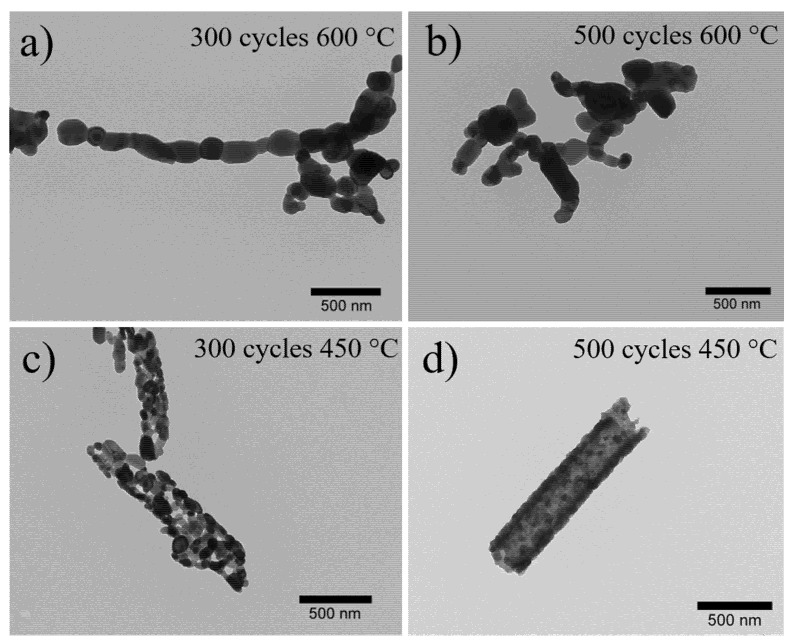
TEM images of ZnO nanostructures obtained by applying different conditions of number of cycles and temperature of calcination: (**a**) 300 cycles and 600 °C; (**b**) 500 cycles and 600 °C; (**c**) 300 cycles; and (**d**) 500 cycles and 450 °C.

The morphologies of ZnO nanostructures obtained following different conditions during ALD and polymer removal processes were studied using TEM and SEM microscopies. Successful zinc oxide hollow nanotubes, from now on called ZnO_NT_, were developed by the calcination process at 450 °C of deposited fibers during 500 cycles of deposition. The resulting images revealed that the number of ALD cycles (300 and 500) and the temperature of calcination (Process A: 450 °C and 600 °C) were the main parameters to optimize the development of homogeneous hollow ZnO nanotubes. Figure 1 shows TEM images of resulting nanostructures by applying these conditions. The resulting morphologies distinguished that the calcination temperature was a critical parameter that determined the formation of the nanotubular structure. When the calcination temperature occurred at 600 °C, the system failed to retain the shape of a nanotube for both numbers of cycles, forming chains of ZnO nanoparticles (see Figure 1a,b). This necklace-like morphology has been previously observed for structures subjected to high calcination temperatures [32,34]. The increase in calcination temperature produced an increase in the size of the ZnO grains from 45 nm for 450 °C to approximately 200 nm for 600 °C. This abrupt change in grain size was primarily responsible for the collapse of tubular morphology. On the other hand, the application of a higher calcination temperature produced a more aggressive thermal decomposition of the polymer, causing fractures of the nanotubes due to the release of compounds (water and acetaldehyde by-products) [31].

Therefore, both the increase in grain size and the thermal decomposition of the polymer contributed to the shape of the nanotubes collapse at 600 °C. On the other hand, when the calcination occurred at 450 °C, the tubular morphology was maintained (Figure 1c,d).

**Figure 2:**
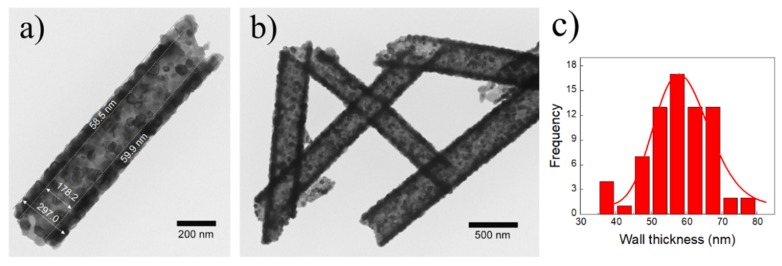
TEM images of ZnO_NT_ (500 ALD cycles and 450 °C as calcination temperature) at different magnifications: (**a**) 10 kx and (**b**) 20 kx; and (**c**) ZnO_NT_ wall thickness histogram.

It is interesting to highlight that although the samples obtained through 300 and 500 ALD cycles exhibited a tubular shape, when they were thermal treated at 450 °C, the sample with 500 ALD cycles presented the formation of a wall with a highly uniform coating (Figure 1d). Based on the aforementioned results, the optimal values for ZnO_NT_ synthesis were fixed at 500 ALD cycles and 450 °C as the number of cycles and the temperature for polymer removal, respectively. Figure 2a,b confirmed that the resulting nanotubes at these conditions exhibited an average wall thickness of 59.5 ± 7.9 nm with an internal diameter of approximately 178.2 nm. The corresponding histogram of wall thickness is presented in Figure 2c.

**Figure 3:**
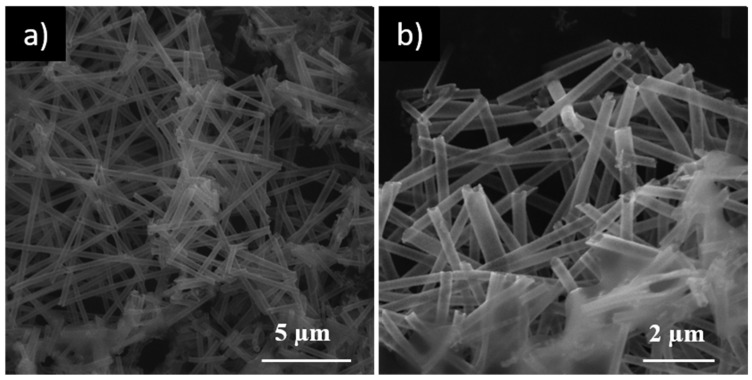
SEM micrographs of ZnO_NT_ at magnifications: (**a**) 10 kx; and (**b**) 20 kx.

ZnO_NT_ were also analyzed through SEM microscopy, and, as Figure 3a,b show, ZnO_NT_ presented a highly homogeneous morphology and hollow nature (additional SEM images are presented in Figure S1 in the Supplementary Material).

The alternative polymer removal process through washing with distilled water at 90 °C (Process B) did not present satisfactory results. The stirring of deposited fibers under hot distilled water allowed a partial dissolution of the inner polymer, and the resulting polymer rate removal was very low, as the TGA results confirmed (next Section 3.3). Nevertheless, interesting TEM images of the sample that resulted from this washing process, (ZnO-PV_f_)-B, are shown in Figure S2 in the Supplementary Material.

### 3.2. Structural Analysis of Nanostructures

**Figure 4:**
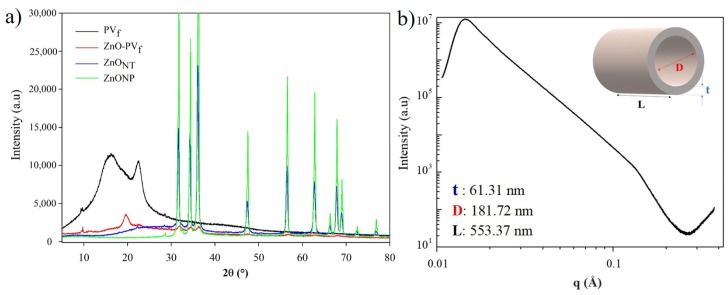
(**a**) XRD diffraction patterns of electrospun polyvinyl alcohol (PV) fibers (PV_f_), coated nanofibers (ZnO-PV_f_), hollow zinc oxide nanotubes (ZnO_NT_), and commercial zinc oxide nanoparticles (ZnONP); (**b**) I(q)-q plot of ZnO_NT_ structure obtained by small-angle X-ray scattering (SAXS).

Figure 4 shows the diffractograms of the structures during the obtaining process of the hollow ZnO_NT_. Although the electrospinning process reduces polymer crystallinity, electrospun PV_f_ demonstrated a semi-crystalline nature through the presence of a sharp peak at 2Ɵ = 22.5°, which is characteristic of a monoclinic PV crystal structure [35]. The deposition of this metal oxide over PV nanofibers (sample ZnO-PV_f_) enhanced this polymer crystallinity, and a new characteristic PV crystalline peak was evidenced at 19.5° [31,35,36]. Probably, the electrospun PV fibers exposition to the ALD chamber temperature (150 °C) and further cooling allowed the reorganization of PV polymeric chains. Furthermore, new peaks related to zinc oxide appeared at 2Ɵ = 31.8, 34.4, 36.2, 56.7 and 62.9, confirming the deposition of this metal oxide by ALD. After the calcination process, these peaks have definitely increased their intensities, and other ZnO characteristic peaks were present at 2Ɵ = 47.6, 66.3, 67.8, 69.1, 72.4, and 76.9°. This diffractogram has clearly shown evidence that hollow ZnO_NT_ presented the hexagonal wurtzite crystal structure [34,37] as commercial ZnO nanoparticles. The principal peaks of both ZnO nanostructures correspond to a quite pure hexagonal stage wurtzite (Joint Committee on Powder Diffraction Standards JCPDS card No. 89-1397). The thermal treatment of calcination not only allowed the total PV polymer removal but also this resulting crystal structure. Sharp and narrow diffractive peaks indicated the efficient crystallization process.

Small-angle X-ray scattering (SAXS) allows the measurement of the scattering behavior at smaller angles. This analysis results assumed a core–shell cylinder with a thickness of 61.31 nm and a radius of 181.72 nm, confirming the measurements obtained through TEM and SEM images.

### 3.3. Thermogravimetric Analyses of Nanostructures

**Figure 5:**
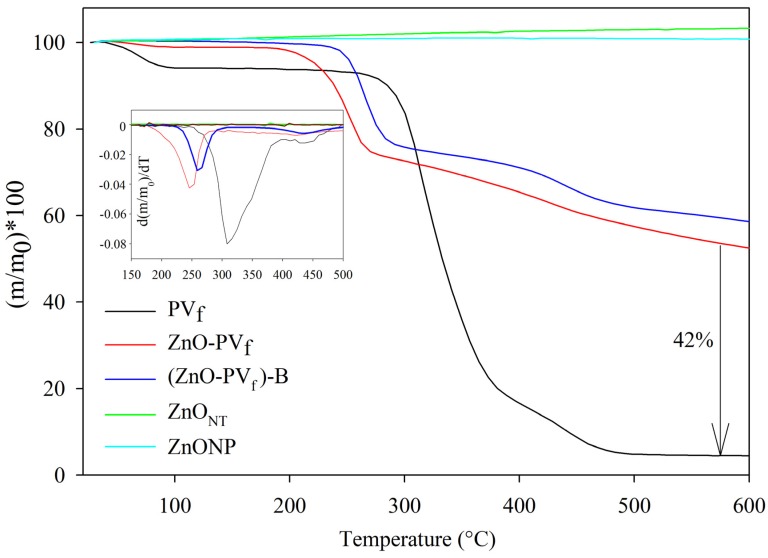
Weight loss with temperature of developed nanostructures (insert: derivative of weight loss with respect to temperature).

Each process for obtaining hollow ZnO_NT_ was analyzed through thermogravimetric analysis, and the TGA curves are shown in Figure 5. The mass loss with respect to temperature of PV_f_, deposited ZnO-PV_f_, and (ZnO-PV_f_)-B provided evidence of the characteristic degradation process of PV polymer in three stages: (i) the dehydration of samples below 100 °C; (ii) the separation of side groups of PV polymer and the loss of hydrogen in chains of the polymer and oxygen bond in C-O groups between 220 and 390 °C; and (iii) the degradation of the principal polymeric chain between 390 and 480 °C [38,39]. After the dehydration process, deposited nanofibers ZnO-PV_f_ and (ZnO-PV_f_)-B presented a decomposition temperature onset lower than PV_f_ nanofibers at approximately 180 and 220 °C, respectively. This fact was probably due to the catalytic nature of ZnO that anticipated PV decomposition [40].

TGA analysis of ZnO-PV_f_ also revealed the ALD-deposited process on PV nanofibers resulted in ZnO of 41.2 wt. % approximately, and the PV removal process through washing [sample (ZnO-PV_f_)-B] was not effective because only 12.3 wt. % of polymer was removed. A previous work based on the development of TiO_2_ nanotubes at the same ALD process conditions presented similar results, which provided evidence of the efficiency and reproducibility of method independently of the type of metal oxide precursors used [31]. TGA analysis was also used to confirm the total elimination of the PV polymer after the calcination and obtaining the hollow ZnO_NT_ nanotubes. This result was evidenced because ZnO_NT_ presented the same TGA curve as commercial ZnONP, demonstrating that ZnO_NT_ nanostructures were PV polymer-free.

### 3.4. Morphological Analysis of Bilayer Nanocomposites Containing ZnO_NT_

**Figure 6:**
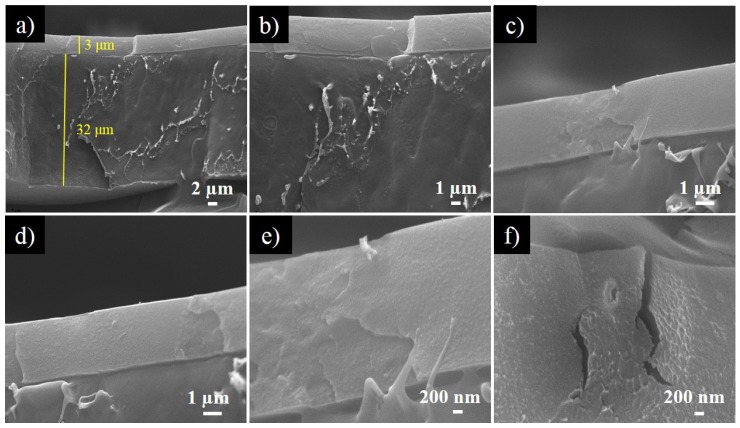
SEM micrographs of bilayer composite 1ZnO_NT_-Acry/PE at different magnifications: (**a**) 5 kx; (**b**) 10 kx; (**c**,**d**) 20 kx; (**e**,**f**) 50 kx.

Figure 6 shows SEM micrographs of the cross-section of the bilayer structure 1ZnO_NT_-Acry/PE at different magnifications. Both layers with their corresponding thickness can be clearly evidenced in Figure 6a, and the effectiveness of the coating process in order to obtain a homogeneous second layer was confirmed. The images indicated that the antimicrobial coating containing ZnO_NT_ presented a smooth surface and a thickness of approximately 3–4 μm. The application of this acrylic polymeric coating containing ZnO_NT_ over the PE substrate allowed the development of an antimicrobial material by incorporating activity in the contact layer. This bilayer system reduces the necessary amount of the antimicrobial ZnO_NT_ to provide the functional activity [41].

The presence of the hollow ZnO_NT_ was not greatly observed due to the low concentration of these nanostructures. Nevertheless, Figure 6e shows a particle that could be a nanotube due to its dimensions, and Figure 6f seems to present a hole derived from the absence of a nanotube after film fracture.

### 3.5. Antibacterial Activity Results

Table 1 shows antibacterial results of active bilayer films expressed in log reduction of cells/cm^2^. Bilayer structures of Acry/PE containing ZnO_NT_ or ZnONP have shown antibacterial activities against the evaluated microorganisms. Antimicrobial activity increased as the concentration of nanostructures increased, and ZnO_NT_-Acry/PE materials presented better antimicrobial activity against both bacteria compared with ZnONP-Acry/PE films. According to JIZ Z 2801:2000, an antimicrobial activity of R > 2.0 log cells/cm^2^ is required to demonstrate the antimicrobial efficacy of active materials [42]. Except for the coating 1ZnONP-Acry against *S. aureus*, both antimicrobial coatings with 1 wt % of ZnO_NT_ or ZnONPs presented a log reduction > 2.0, demonstrating the great antimicrobial activity of this metal oxide. Specifically, the bilayer structure 1ZnO_NT_-Acry/PE resulted in the total inhibition of *E. coli*. Several mechanisms have described ZnO antimicrobial activity, including cell damage through their interaction with the microorganisms, ions release from zinc oxide, and the reactive oxygen species (ROS) formation by the activation of this metal oxide [43,44]. In this case, the possible mechanism could be the interaction of ZnO_NT_ and ZnONP with the bacteria due to the conditions of antimicrobial assay. The antimicrobial activity efficacy of zinc oxide nanoparticles is influenced by morphology, size, and surface energy. The morphology of ZnO nanoparticles is greatly determined by synthesis conditions such as solvents, temperature, pH, and precursor types, which are employed in precipitation, sol–gel, and solvothermal techniques in order to obtain circular, tubular, or irregular morphologies [13,45]. In this study, ZnO_NT_ morphology, which had a high aspect ratio due to their hollow structure and wall thickness, was controlled by electrospinning and ALD parameters. The high aspect ratio of ZnO_NT_ allowed exhibiting a strong antibacterial activity against Gram-positive and negative bacteria compared to ZnONP. A similar result was obtained with hollow titanium dioxide nanotubes against *Escherichia coli*. In this study, a concentration of TiO_2_ nanotubes lower than TiO_2_ nanoparticles was required to obtain a total reduction of Gram-negative bacteria.

In the case of materials with ZnONP-containing coating, the antimicrobial activity was similar against both bacteria, and the structure 1ZnONP-Acry/PE showed evidence of the highest activity against *E. coli*, with a log reduction of 2.32 cells/cm^2^. Both films presented higher effectiveness against Gram-negative bacteria.

On the other hand, the highest antimicrobial activity against *S. aureus* was obtained with 1ZnO_NT_-Acry/PE film with a log reduction of 2.46 cells/cm^2^. The lower antimicrobial activity of nanostructures against *S. aureus* is due to its intracellular antioxidant content as well as the presence of potent detoxification agents [10]. Additionally, the difference of the cell wall structure to both bacteria plays an important role in the antimicrobial activity of nanoparticles, which had been observed with other metal oxides [46]. Gram-negative bacteria have a thin peptidoglycan layer that facilitate the mobility of metal ion nanoparticles to the cell and assist the interaction between ZnO nanoparticles with bacterial cell walls, while Gram-positive bacteria have a thick layer of peptidoglycan that protect and cause more resistance. In addition, the negative charge of the lipopolysaccharide layer in Gram-negative bacteria leads to intracellular damages and the destruction of proteins and DNA [47].

Generally, the results showed a high antibacterial activity in films coated with ZnO_NT_. The concentration, size, and shape of hollow ZnO nanotubes directly influenced the antimicrobial efficacy of zinc oxide, where a larger surface area and higher concentration of ZnO nanotubes in the PE-NT were responsible for the highest antibacterial activity [13].

### 3.6. Antiviral Activity against Norovirus Surrogate

In the current study, FCV, a norovirus surrogate broadly used for testing disinfectants (EPA (Environmental Protection Agency), 2002) and virucidal films [48,49,50], was exposed to ZnO-containing films for 24 h following the ISO 22196:2011. As Table 2 shows, unfortunately, none of these evaluated nanocomposites showed antiviral activity under the experimental conditions tested. FCV concentrations control and in contact with nanocomposites did not present significant differences. Currently, some metallic nanoparticles as silver and copper with demonstrated virucidal activity have been used to develop antiviral polymers. Specifically, Martínez et al. (2013) have applied silver-infused polylactide (PLA) films for the inactivation of FCV [49]. The antiviral activity of silver-PLA films was dose-dependent, where increasing concentrations of silver showed an increased reduction in viral titers. Nevertheless, as observed in this study, FCV was less susceptible than *Salmonella*, suggesting a higher resistance of viruses to antimicrobial compounds than bacteria. In line, micrometer-sized magnetic hybrid colloid activated with AgNPs reduced the infectivity of murine norovirus, which is another norovirus surrogate, by more than 2 log, while more than a 6 log reduction was reported for *E. coli* [50,51]. Similarly, AgNP-containing fiber mats reduced MNV (novel mouse norovirus) infectivity by only 0.86 log while under the same conditions, and no viable counts of *Salmonella enterica* and *Listeria monocytogenes* were recorded [52].

## 4. Conclusions

Hollow zinc oxide nanotubes with an average diameter of 180 nm and thickness of approximately 60 nm were successfully obtained through the combination of electrospinning and atomic layer deposition processes. A total of 500 ALD cycles of diethyl zinc and water as precursors over electrospun polyvinyl alcohol fibers followed by a calcination process at 450 °C resulted in homogenous hollow zinc oxide nanotubes. Subsequently, bilayer systems composed by an acrylic polymeric coating containing as-synthesized zinc oxide nanotubes over a polyethylene substrate were successfully developed. Antimicrobial analysis revealed that the antimicrobial effectiveness was dependent on zinc oxide concentration and the antimicrobial materials presented the highest activity against Gram-negative bacteria. When compared to a bilayer system containing commercial zinc oxide nanoparticles, materials with zinc oxide nanotubes presented higher antimicrobial effectiveness probably because their tubular morphology presented a higher specific surface area and lower aggregation than commercial spherical zinc oxide nanoparticles.

## Figures and Tables

**Table 1:** Antibacterial results of coated PE containing ZnO_NT_ and ZnONP at 0.5 and 1 wt. % against different microorganisms.

Microorganism	*Escherichia coli*		*Staphylococcus aureus*	
Sample	Cell Conc. cells/cm^2^	Log Reduction	Cell Conc. cells/cm^2^	Log Reduction
PE	4.69 × 10^4^	-	5.28 × 10^5^	-
0.5ZnO_NT_-Acry/PE	2.22 × 10^2^	2.32	2.35 × 10^4^	1.35
1ZnO_NT_-Acry/PE	0.00	4.67	1.85 × 10^3^	2.46
0.5ZnONP-Acry/PE	1.08 × 10^4^	0.64	1.07 × 10^5^	0.69
1ZnONP-Acry/PE	3.13 × 10^1^	3.18	2.80 × 10^4^	1.27

**Table 2:** Antiviral effect of ZnO films on feline calicivirus (FCV) titers (log TCID_50_/mL) after overnight incubation at 25 °C at 100% relative humidity (RH) following ISO 22196:2011. PE: polyethylene, TCID_50_: tissue culture infectious dose

	High FCV Concentration		Low FCV Concentration	
Sample	Log TCID_50_/mL	Reduction	Log TCID_50_/mL	Reduction
PE	6.66 ± 0.29		3.91 ± 0.38	
0.5ZnO_NT_-Acry/PE	6.57 ± 0.22	0.08	3.78 ± 0.19	0.13
1ZnO_NT_-Acry/PE	6.70 ± 0.25	−0.04	4.03 ± 0.19	−0.13
0.5ZnONP-Acry/PE	6.57 ± 0.25	0.08	3.53 ± 0.07	0.38
1ZnONP-Acry/PE	6.24 ± 0.38	0.42	3.53 ± 0.14	0.38

## Supplementary Materials

The following are available online at https://www.mdpi.com/2079-4991/10/3/503/s1, Figure S1. SEM micrographs (3D-SEM) of hollow ZnO_NT_ at magnifications: (**a**) 15 kx; and (**b**) 10 kx; Figure S2. TEM images of ZnO-PV_f_ after washing process: (ZnO-PV_f_)-B; and Figure S3. TEM image of commercial ZnONP.
