# Supplementary material for: Antimicrobial Bilayer Nanocomposites Based on the Incorporation of As-Synthetized Hollow Zinc Oxide Nanotubes
Source: Nanomaterials (Basel). 2020 Mar 11;10(3):503. doi: 10.3390/nano10030503 (PMC7153247; doi:10.3390/nano10030503)
Supplement: Supplementary file 1 [file nanomaterials-10-00503-s001.pdf]

Supplementary Material

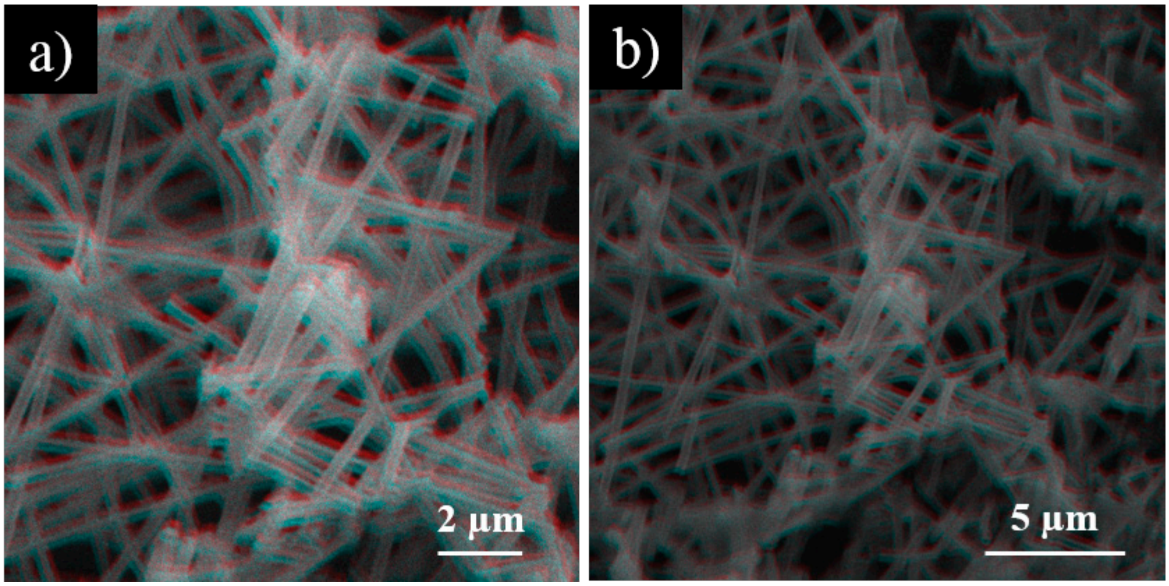

Fig. S1. SEM micrographs (3D-SEM) of hollow ZnO_{NT} at magnifications: a) 15 kx; and b) 10 kx.

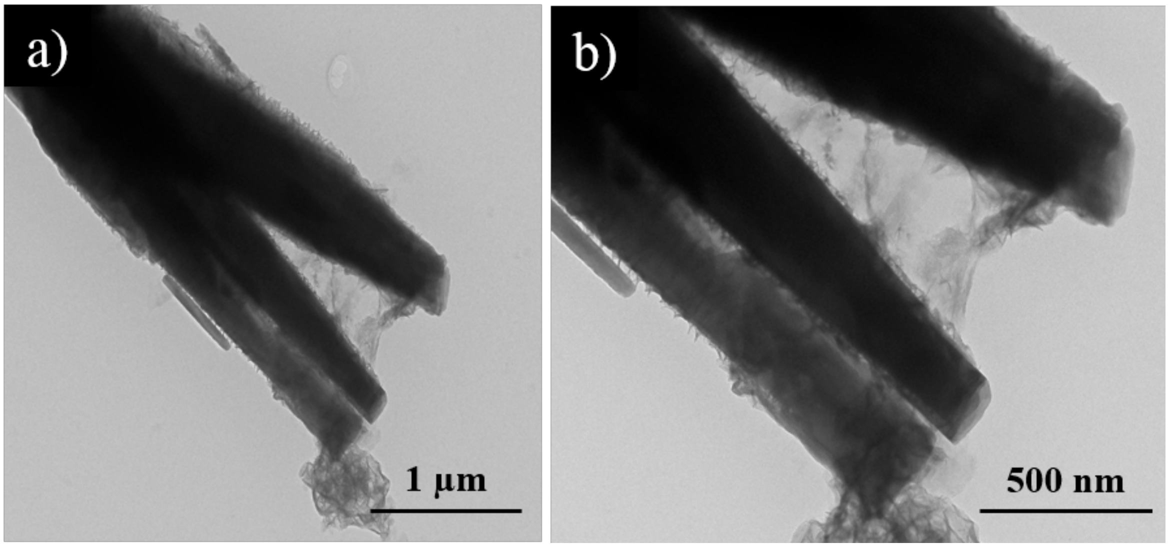

Fig. S2. TEM images of ZnO-PV_f after washing process: (ZnO- PV_f)-B.

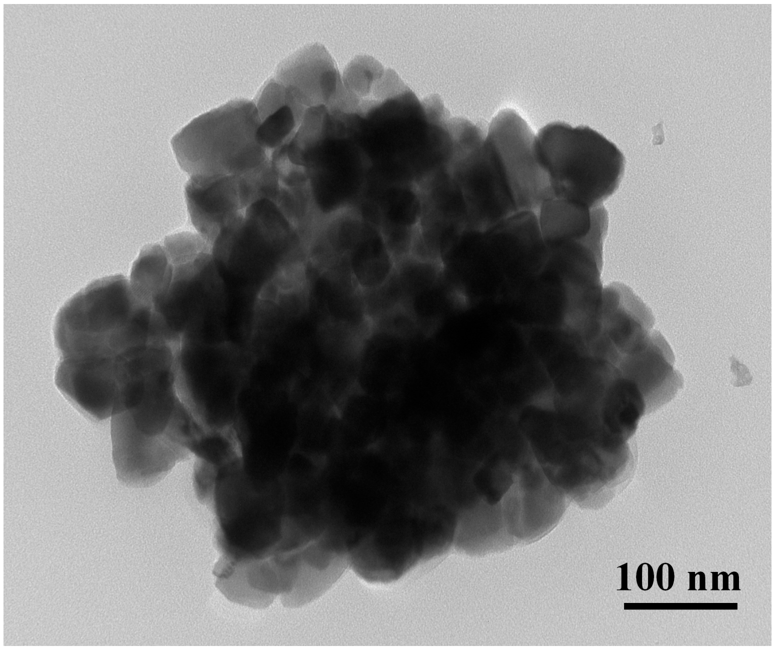

Fig. S3. TEM image of commercial ZnONP.
